# Health Implications of an Immigration Raid: Findings from a Latino Community in the Midwestern United States

**DOI:** 10.1007/s10903-016-0390-6

**Published:** 2016-04-04

**Authors:** William D. Lopez, Daniel J. Kruger, Jorge Delva, Mikel Llanes, Charo Ledón, Adreanne Waller, Melanie Harner, Ramiro Martinez, Laura Sanders, Margaret Harner, Barbara Israel

**Affiliations:** 1 Department of Health Behavior and Health Education, School of Public Health, University of Michigan, 1415 Washington Heights, Ann Arbor, MI 48109 USA; 2 School of Social Work, University of Michigan, Ann Arbor, MI USA; 3 Department of Family Medicine, University of Michigan Medical School, Ann Arbor, MI USA; 4 Washtenaw County Community Health Worker, Ann Arbor, MI USA; 5 Washtenaw County Public Health Department, Ypsilanti, MI USA; 6 Washtenaw Interfaith Coalition for Immigrant Rights (WICIR), Ypsilanti, MI USA; 7 School of Social Work, WICIR, University of Michigan, Ann Arbor, MI USA

**Keywords:** Immigration policy, Latino, Community health, Mixed status, Undocumented, Immigrant

## Abstract

Immigration raids exemplify the reach of immigration law enforcement into the lives of Latino community members, yet little research characterizes the health effects of these raids. We examined the health implications of an immigration raid that resulted in multiple arrests and deportations and occurred midway through a community survey of a Latino population. We used linear regression following principal axis factoring to examine the influence of raid timing on immigration enforcement stress and self-rated health. We controlled for age, sex, relationship status, years in the county in which the raid occurred, children in the home, and nativity. 325 participants completed the survey before the raid and 151 after. Completing the survey after the raid was associated with higher levels of immigration enforcement stress and lower self-rated health scores. Findings indicate the negative impact of immigration raids on Latino communities. Immigration discussions should include holistic assessments of health.

## Introduction

Undocumented immigrants experience a pervasive fear of deportation that negatively impacts their psychological, emotional, and physical health [1–4]. The possibility of immigration status disclosure—which could lead to deportation—decreases health care and social service utilization [2, 4–7]. Local police collaboration with federal immigration law enforcement exacerbates distrust between local law enforcement and community members, decreasing the likelihood that undocumented immigrants will report crimes, even if they are the victims [8–10]. Constant surveillance and scrutiny of immigration status transforms public space into an area of heightened risk, and undocumented immigrants become less likely to walk their children to school, visit parks or the grocery store, and participate in a range of sociocultural activities [11–14]. Taken together, a growing body of research illustrates the direct and indirect effects of immigration law enforcement and policy on the health of undocumented immigrants and the communities in which they live.

Quasi-experimental studies have considered shifts in health-related behaviors after immigration policy change. Findings suggest that policies requiring individuals to provide evidence of immigration status limit access to resources, whether or not individuals are undocumented. For example, after the passage of the Alabama Taxpayer and Citizen Protection Act, which required proof of citizenship to receive public benefits, Latinas were less likely to utilize county health services. This included services exempt from residency requirements, such as treatment for STIs [15, 16]. Similarly, following the passage of Arizona Senate Bill 1070 (SB 1070), which gave police the power to detain individuals who could not verify citizenship, Mexican-origin adolescent mothers were less likely to use public assistance or take their infants to receive medical care [17]. SB 1070 also increased fear of immigration enforcement, restricted neighborhood mobility, and decreased trust in law enforcement, with impacts on health [11].

These quasi-experimental studies present strong evidence of the immediate repercussions of immigration policy on health. However, a focus on state level policies in states with omnibus anti-immigrant legislation omits the daily immigration enforcement events that are ubiquitous in mixed status immigrant communities [18] but are not often covered by mainstream media. Immigration home raids, in which Immigration and Customs Enforcement (ICE) agents enter a residence to detain undocumented immigrants, represent extreme examples of immigration law enforcement with emerging evidence of their influence on health [19–21]. A typical home raid occurs in the pre-dawn hours when occupants are sleeping. Agents, often with guns drawn and wearing body armor, surround the house, pound on doors, and enter the home after receiving consent, though reports of consent differ [22]. Other individuals suspected of being undocumented may be taken as “collateral” arrests, a process rooted in racial profiling [23]. These arrests frequently include witnesses, often children [10, 14, 19, 20, 22, 24–30]. Despite the effects of these raids on health however, they have received scant attention in the health literature, often mentioned as one of many aspects of increased immigration enforcement [11].

The current study addresses this gap in the literature by building upon the quasi-experimental designs of previous public health work to investigate the influence of an immigration raid on the health of a Latino/a (henceforth: Latino) community in the Midwestern US. We consider the effects of an immigration raid on a de facto mixed-use property, in which multiple individuals were arrested and deported. Three months before the raid, we began data collection for the *Encuesta Buenos Vecinos* (EBV; “Good Neighbors Survey”) to investigate the social determinants of health among Latinos in the county. The survey data thus provide a unique opportunity for a natural experiment in which we investigate the effects of a federal/local immigration raid on health in a state that has not passed omnibus anti-immigrant policy (Michigan). Understanding these effects may better prepare health professionals to respond to crises and mitigate the damage to health that occurs following immigration enforcement.

## Methods

Washtenaw County, MI, has a population of over 12,000 Latinos [31] (about 3.5 % of the population), yet Latinos are frequently underrepresented in county-level data [32]. To gain a more accurate depiction of the health of the Washtenaw County Latino population, collaborators at the University of Michigan, Washtenaw County Public Health Department, and Casa Latina, a community-based organization engaged with the local Latino community, designed and implemented the EBV. Community partners were involved in the creation and review of the EBV instrument, and feedback following the pilot phase was used to shape survey implementation. The University of Michigan’s Institutional Review Board approved the research.

The EBV queried a range of topics, including health-related knowledge, attitudes, beliefs, and behaviors; access to health care; sources of health information; family and child health; and social and neighborhood conditions [32]. Although the study was not designed to capture the effects of immigration raids specifically, it did aim to address issues of concern related to immigration and immigration law enforcement.

The survey was conducted in Spanish or English, online or face-to-face, by trained members of the local Latino community. Inclusion criteria included being 18 years of age or older, identifying as “Hispanic or Latino/a,” and living in Washtenaw County. A total of 487 Latinos participated in the survey from September 2013 to January 2014. An immigration raid occurred about halfway through the data collection period, resulting in 325 participants who completed the survey before and 151 who completed the survey after the raid.

### The Raid

On the evening of November 7, 2013, a joint task force composed of agents from ICE and the County Sheriff’s Department conducted an immigration raid on a two-story warehouse unit not zoned for residential use that was converted into a mechanic shop on the first floor and living quarters on the second floor. The Sherriff’s Department utilized a special weapons and tactics (SWAT) unit because of the belief that firearms and drugs may be present in the facility. The mechanic shop was well known to and utilized by many local residents up to the day of the raid.

Several members of a local immigration advocacy organization, the Washtenaw Interfaith Coalition for Immigrant Rights (WICIR [19]), were notified by members of the community of the raid, which was preceded by ongoing arrests of Latinos throughout the day, and arrived on the scene to witness events and support the individuals present. By multiple accounts, including testimony of those in the facility when the raid occurred, WICIR volunteers present immediately after the raid, and information obtained through a Freedom of Information Act request, three women, four children under the age of five, and multiple men were in the facility when it was raided. Agents pushed in a door, breaking the lock, and ordered the women to the floor at gunpoint while two were holding infants in their arms. Multiple men were detained and later deported. Three days later, approximately 40 community members, including the women involved in the raid and multiple authors of the current study, attended a community gathering at a local church. The women described their treatment by officers and their fears of the psychological repercussions that witnessing the raid could have on their children. Community members and advocates agreed to re-engage the Sheriff’s office in order to discuss how to rebuild trust between the community and local police.

### Measures

#### *Independent variable: Raid timing*

We created an independent variable (raid timing) based on whether participants completed the EBV before or after the day of the raid (0 = before, 1 = after). The EBV includes data from 487 completed surveys. We excluded five participants whose surveys were completed on the day of the raid and six participants whose completion date was unknown. The analyses represent 476 individuals, 325 participating before the raid and 151 afterward.

#### *Immigration enforcement stress and self*-*rated health (SRH)*

We utilized three survey measures originally intended to capture day-to-day stress related to immigration enforcement: “My legal status has limited my contact with family and friends,” “I will be reported to immigration if I go to a social service agency,” and “I fear the consequences of deportation.” The first two items were adapted from the acculturative stress scale of the 2012 National Latino and Asian American Study [33], and the third was created by the EBV team. Participants responded to each item on a scale from 1 (strongly disagree) to 5 (strongly agree). Thus, higher scores (more agreement) represent a stronger negative influence of immigration enforcement on participants’ day-to-day lives.

Secondly, we utilize a measure of self-rated health to which participants responded on a scale from 1 (poor) to 5 (excellent). SRH has been shown to be strongly and consistently associated with mortality [34], with even stronger associations in recent decades [35]. Conceptually, SRH is proposed to capture a state of “the human body and mind” (p. 307) that takes into consideration physiological sensations, comparison groups, and health behaviors. More pertinent to the current study, research has considered how SRH is linked to social capital (such as trust among neighbors or norms of reciprocity) [36], with lower SRH scores related to less social capital. Associations between SRH and mortality have been found in Latino subsamples, though the relationships appear to be moderated by acculturation [37] and language [38].

#### *Demographics*

We use standard demographic variables including sex (0 = female), age in years (continuous), and relationship status (0 = not in a relationship) as well as variables that assess language ability (English communication skills, language spoken with friends, and language spoken with family) and nativity (0 = born outside of the US). Lastly, we include two variables that assess participant ties to the community where the raid occurred, including years in Washtenaw County and presence of children in the home (0 = no). Immigration status was not directly assessed because of concerns for the vulnerability of survey participants.

### Analysis

Pairwise correlation was used to examine the relationships among the three immigration enforcement stress measures, SRH, sex, nativity, the presence of children in the home, and raid timing. We utilized two linear regression models with (1) the intensity of immigration enforcement stress, and (2) self-rated health as outcome variables. To create the outcome variable for the first model, we used principle axis factoring of the three immigration enforcement stress measures to create a single factor that captures the intensity of immigration enforcement on participants’ day-to-day lives. We control for age, sex, relationship status, years in Washtenaw County, children in the home, and nativity.

## Results

Table 1 presents characteristics for the sample in full and stratified by raid timing. Fully 57.4 % of participants were female and the mean age was 36.6 years (SD = 13.7 years). The mean length of US residence was 15.3 years (SD = 10.9 years) and of county residence was 9.1 years (SD = 7.6 years). The mean self-rated health score was 3.6 (SD = 1.1), or between “good” and “very good.” Participants were more likely to speak Spanish than English with friends and with family. Over half of participants had children under the age of 18 in their homes. The majority of the sample (84.3 %) was born outside of the US, mostly in Mexico (36.8 %) or Central America (24.4 %) (not shown). While the recruitment strategy did not change, there were differences in the study sample before and after that raid. Those who completed the survey after the raid were younger by about 5.5 years and were less likely to be foreign-born (Table 1). Nonparametric tests showed that participants who completed the survey after the raid were also more likely to participate on-line (vs. hard copy), *Z* = 4.29, *p* < 0.001, and in English (vs. Spanish), *Z* = 2.01, *p* = 0.045.

**Table 1:** Sample characteristics of EBV participants N = 476

Measure	Full sample	Before raid	After raid	X^2^/t value, *p* value
Women (%)	57.4	60.0	51.7	2.9, *p* = 0.09
Age, years (SD)	36.6 (13.7)	38.4	32.9	4.10, *p* < 0.001
Education, years (SD)	12.0 (4.4)	12.2	11.6	1.37, *p* = 0.17
Time in US, years (SD)	15.3 (10.9)	15.3	15.3	0.00, *p* = 1.0
Time in Washtenaw County, years (SD)	9.1 (7.6)	9.9	7.5	3.19, *p* < 0.01
Self-rated health, mean (SD)	3.6 (1.1)	3.6	3.4	1.9, *p* = 0.06
Born abroad (%)	84.3	87.2	78.2	6.0, *p* = 0.01
Children in home (%)	54.4	55.9	51.4	0.8, *p* = 0.36
In a relationship (%)	62.9	63.3	62.0	0.08, *p* = 0.78
Language factors, mean (SD)				
How well do you speak Spanish? (1-not at all to 6-excellent)	5.2 (1.2)	5.2	5.0	1.61, *p* = 0.12
How well do you speak English? (1-not at all to 6-excellent)	3.9 (1.7)	4.0	3.7	1.71, *p* = 0.09
Language spoken with family				1.77, *p* = 0.41
Spanish (%)	75.2	75.8	74.0	
English (%)	12.0	10.7	14.7	
Both English and Spanish (%)	12.8	13.5	11.3	
Language spoken with friends				2.1, *p* = 0.35
Spanish (%)	52.3	51.7	53.6	
English (%)	26.0	24.8	28.5	
Both Spanish and English (%)	21.7	23.5	17.9	

*T* test used for continuous data, Chi-square tests used for categorical data

### *Pairwise correlation analysis*

Immigration enforcement stress measures correlated strongly, and were significantly higher after the raid, for those born abroad and for those with children in the home (Table 2).

**Table 2:** Pairwise correlation analysis of immigration related health and demographic factors and raid timing

	1.	2.	3.	4.	5.	6.	7.
1. My legal status has limited my contact with family and friends^a^	1						
2. I fear the consequences of deportation^a^	0.74***	1					
3. I will be reported to immigration if I go to a social service agency^a^	0.76***	0.72***	1				
4. Self-rated health^b^	−0.26***	−0.27***	−0.24***	1			
5. Sex (1 = male)	0.04	−0.01	0.06	0.09*	1		
6. Nativity (1 = born in the US)	−0.32***	−0.29***	−0.26***	0.13**	0.01	1	
7. Children in house (1 = yes)	0.20***	0.20***	0.12*	−0.08	−0.05	−0.26***	1
8. Raid timing (1 = after)	0.11*	0.12**	0.04	−0.09	0.08	0.11*	−0.04

* *p* < 0.05; ** *p* < 0.01; *** *p* < 0.00

^a^1 = strongly disagree, 5 = strongly agree

^b^1 = poor, 5 = excellent

### *Multivariate models*

Principle axis factoring of the three immigration enforcement stress measures resulted in one factor with an Eigenvalue of 2.1, with each item having a factor loading greater than 0.8. We consider this factor to be a measure of the intensity of immigration enforcement stress. Linear regression of this factor while controlling for age, sex, relationship status, years in the county, children in the home, and nativity indicated that raid timing was significantly associated with immigration enforcement stress such that those who completed the survey after the raid reported higher levels of immigration enforcement stress (Table 3: model 1). Having children in the home and being born outside of the US were associated with higher levels of immigrant enforcement stress. The second model indicated that raid timing was also significantly associated with self-rated health. That is, completing the survey after the raid was associated with a 0.4 unit decline in SRH (Table 3: model 2).

**Table 3:** Regression analysis of raid timing on intensity of immigration enforcement and self-rated health

	B (SE)	Significance
*Model 1: Immigration enforcement stress intensity (n* *=* *429)*		
Raid timing (1 = after)	0.29 (0.09)	<0.01
Age	−0.00 (0.00)	0.22
Sex (1 = male)	0.06 (0.09)	0.47
Relationship status (1 = in a relationship)	−0.16 (0.09)	0.10
Years in Washtenaw County	0.01 (0.01)	0.28
Children in house (1 = yes)	0.25 (0.09)	<0.01
Nativity (1 = born in the US)	−0.78 (0.12)	<0.001
*Model 2: Self rated health (n* *=* *443)*		
Raid timing (1 = after)	−0.41 (0.10)	<0.001
Age	−0.03 (0.00)	<0.001
Sex	0.17 (0.10)	0.07
Relationship status (1 = in a relationship)	0.04 (0.11)	0.69
Years in Washtenaw County	0.01 (0.01)	0.40
Children in house (1 = yes)	−0.07 (0.11)	0.49
Nativity (1 = born in the US)	0.21 (0.14)	0.12

## Discussion

The current natural experiment demonstrates the impact of an immigration raid on the health of a Latino community in the Midwestern US. Results show that participants who completed the EBV survey after the raid reported higher levels of immigration enforcement stress and lower SRH scores, despite a lower percentage of foreign-born participants completing the survey after the raid. Our analyses present strong evidence of the negative influence of immigration raids both directly and indirectly on the lives and health of Latinos in the communities in which raids occur.

Previous studies noted that deportation fear is related to psychological and emotional distress and lower SRH scores [1, 2]. Although we did not explicate the mechanism by which a raid could influence SRH, we found that participants who completed the survey after the raid reported feeling less free to interact with their social networks, less able to use government services, and increasingly fearful of the consequences of deportation (Table 2). Thus, we suggest that participants would be correspondingly less likely to move about and engage with their communities, reflecting a reduction in social capital [36] that may be related to lower SRH scores. Future research is needed to investigate this mechanism.

Immigration raids, acute events that may have immediate effects on communities, occur within populations who experience an inequitable need for support services and may thus exacerbate health inequities. We recommend that health researchers broaden our conversations about immigration enforcement and its health implications. It is critical that researchers continue to investigate the direct effects of immigration enforcement on the individuals who experience it—such as the depression, anxiety, and trauma engendered by one’s arrest, deportation, or witness to a raid. However, we must also consider how enforcement actions tear at the fabric of US society, alienating immigrant and US born Latinos alike by discouraging them from integration and the utilization of services to which they are entitled.

Lastly, our results indicate that having children in the home and being born outside of the US were associated with higher levels of immigration enforcement stress. Maintaining a healthy community requires access to preventative health services, yet fear of accessing these services may increase in the wake of immigration enforcement actions. Members of the community who may already be marginalized and vulnerable—foreign-born women with children in the home—may be less likely to seek out these services following immigration raids. Thus, community health advocates need to work diligently to ensure separation between immigration enforcement and social service provisioning.

### Limitations

Though this study has a number of strengths, including the natural experiment design and community-based data collection, there are limitations. First, this study is cross-sectional, and thus we are unable to discern if differences in item scores before versus after the raid are the result of within-individual differences. However, quantitative research on immigration raids is extremely challenging—if not impossible—to conduct longitudinally due in part to the purposefully unpredictable nature of these raids. The novel approach used in this study speaks to the importance of community-based collaborations that encourage awareness of community events and existing community data. Secondly, it is not known how well these results generalize to other raids. Although many facets of this raid resemble those reported nationwide [22], including the presence of numerous heavily armored officers who yelled commands and pointed weapons at individuals who were not the targets of the raid, there are differences. Most notably was the use of a SWAT unit who entered the facility without gaining consent, as is required in ICE raids. Also, the building in which the raid occurred was not zoned for residence, but families inhabited it nonetheless. Typical immigration home raids likely occur at traditional homes in residential neighborhoods. However, given the intensity and violence of immigration raids, we argue that the detrimental effects on community and individual health are likely to be similar despite differing environmental circumstances.

Third, we are unable to discern if results are attributable to other events occurring around the same time. However, the advocacy group responding to the raid also staffs an “urgent responder” phone that assists members of the community with immigration related emergencies, and no other calls for events of this magnitude were recorded during this period. We note that immigration reform was heavily discussed in the media at the time, and it is possible that a particular story was salient around the month of the raid. For example, many individuals were possibly aware that the US House of Representatives would be voting on bills that included legislative changes that could affect their or their families’ immigration statuses or increase border militarization. For this reason and others, future research using quantitative and qualitative data is needed to investigate to what extent raids are distinguishable from other immigration law enforcement activities, events, or media discussions.

## Conclusion

This raid was an extreme example of an immigration law enforcement effort that was unpredictable to the community in which it occurred, incorporated overt militarized displays of power, and included an invasion of personal space. Results show that completing the community survey after the raid was associated with higher levels of immigration enforcement stress and lower self-rated health scores. Although novel to the literature, due to the overt violence and trauma of these raids, the results are perhaps not surprising. As immigration policy continues to be a topic of national debate, we must include in these conversations a holistic assessment of the social cost of enforcement policies, including how they may exacerbate existing health inequities among marginalized populations.
